# It Is Worthwhile to Cryopreserve Metaphase I Oocytes for Patients With Oocytes Cryopreservation: A Live Birth Case Report

**DOI:** 10.7759/cureus.89676

**Published:** 2025-08-09

**Authors:** Szu-Yun Niu, Yu-Chiao Yi, Hwa-Fen Guu, Ming-Jer Chen

**Affiliations:** 1 Department of Obstetrics and Gynecology and Women’s Health, Taichung Veterans General Hospital, Taichung, TWN; 2 Division of Infertility, Lee Women’s Hospital, Taichung, TWN

**Keywords:** controlled ovarian hyperstimulation, elective oocyte cryopreservation, immature oocytes, metaphase i oocyte, rescue in vitro maturation

## Abstract

In controlled ovarian hyperstimulation (COH) cycles, many immature oocytes are retrieved and are typically discarded. Our aim is to demonstrate a case of live birth achieved through in vitro maturation (IVM) after thawing of a metaphase I (MI) oocyte in a patient with elective oocyte cryopreservation.

A 36-year-old woman underwent elective oocyte cryopreservation in 2021, retrieving 28 oocytes. In 2023, 12 oocytes were thawed, with only four at metaphase II (MII) oocytes. Rescue IVM was performed on the remaining eight MI oocytes, successfully maturing five to MII. Intracytoplasmic sperm injection (ICSI) resulted in four two pronuclei and two day-five blastocysts, graded 3AA and early blastocyst - both derived from MI oocytes that matured to MII. The 3AA blastocyst was transferred in 2023, leading to the birth of a healthy male infant weighing 3580 g.

This case underscores the developmental potential of immature oocytes in patients undergoing elective oocyte cryopreservation and suggests rescue IVM may serve as a valuable strategy to improve reproductive outcomes.

## Introduction

In controlled ovarian hyperstimulation (COH) cycles, immature oocytes usually account for 15%-30% of the total number of retrieved oocytes. This proportion may even exceed 50% in some patients with advanced age, poor ovarian response (POR) or polycystic ovary syndrome (PCOS) [1]. Rescue in vitro maturation (IVM) can be applied to these immature oocytes to enhance their developmental potential. Rescue IVM is defined as the culture of immature oocytes - typically discarded - collected from conventional superovulation-in vitro fertilization (IVF) or intracytoplasmic sperm injection (ICSI) cycles [2]. Strassburger et al. (2004) demonstrated that two pronucleus fertilization rates varied depending on the maturation status of oocytes: 28% for metaphase I (MI) oocytes arrested in in vitro maturation, 33% for MI oocytes matured in vitro, 44% for rescued MI oocytes, and 68% for metaphase II (MII) oocytes following ICSI. These findings indicate that rescue in vitro maturation (IVM) can improve the fertilization potential of MI oocytes [3]. Similarly, Jeong Hee Moon et al. reported that MI oocytes matured to MII in vitro prior to ICSI exhibited lower developmental competence compared to in vivo matured MII oocytes. This included reduced fertilization rates, blastocyst formation, and euploidy rates. However, euploid blastocysts resulted in similar live birth rates, which indicated that MI oocytes with rescue in vitro maturation can still be useful even though the overall developmental competence was lower than that of their in vivo matured counterparts [4]. Despite these findings, limited studies have explored the role of rescue IVM in patients undergoing oocyte cryopreservation. In this case report, we present a unique case of a patient who achieved a live birth through the oocyte retrieved as MI and matured to MII following eight hours’ culture in G-IVF PLUS medium (Vitrolife, Gothenburg, Sweden). This case highlights the potential value of preserving MI oocytes in patients undergoing cryopreservation, warranting further investigation.

## Case presentation

A 36-year-old woman first presented to our hospital in 2020 for social cryopreservation. She was unmarried, with no history of pregnancy or underlying medical conditions. Her menstrual cycles were regular, with an interval of 32 days and a duration of approximately five days. At the initial visit, her anti-Müllerian hormone (AMH) level was 4.8 ng/mL.

In January 2021, she underwent ovum pick-up (OPU) using a gonadotropin-releasing hormone (GnRH) antagonist protocol for ovarian stimulation. Baseline hormonal levels on cycle day three showed follicle-stimulating hormone (FSH) at 5.55 mIU/mL, luteinizing hormone (LH) at 4.71 mIU/mL, and estradiol at 39 pg/mL(Table 1). She was administered follitropin alfa (Gonal-F) 225 IU and menotropin (Menopur) 75 IU daily from cycle day three to day 12. Cetrorelix (Cetrotide) 0.25 mg was given from cycle day eight to day 12, and ovarian triggering was achieved with triptorelin acetate (Decapeptyl) 0.2 mg on day 12. A total of 28 oocytes were retrieved.

**Table 1:** Hormone Profile: Measured and Reference Values FSH: Follicle-stimulating hormone, LH: luteinizing hormone; HRT: hormone replacement therapy

Hormone profile	Obtained values	Reference
Anti-Müllerian Hormone	4.8 ng/mL	Female (18-25 y/o) : 0.96-13.34 ng/mL; Female (26-30 y/o) : 0.17-7.37 ng/mL; Female (31-35 y/o) : 0.07-7.35 ng/mL; Female (36-40 y/o) : 0.03-7.15 ng/mL; Female (41-45 y/o): 0.00-3.27 ng/mL; Female (≧ 46 y/o): 0.00-1.15 ng/mL
FSH	5.55 mIU/mL	Females: follicular phase: 3.03-8.08 mIU/mL; mid-cycle phase: 2.55-16.69 mIU/mL; luteal phase: 1.38-5.47mIU/mL; postmenopausal: 26.72-133.41mIU/mL
LH	4.71 mIU/mL	Females: follicular phase: 1.80-11.78 mIU/mL; mid-cycle peak phase: 7.59-89.08 mIU/mL; luteal phase: 0.56-14.00 mIU/mL; postmenopausal females without HRT: 5.16-61.99 mIU/mL
Estradiol	39 pg/mL	Females: follicular phase: 21-251 pg/mL; mid-cycle phase: 38-649 pg/mL; luteal phase: 21-312 pg/mL; postmenopausal females not on HRT: <10-28 pg/mL; postmenopausal Females on HRT: <10-144 pg/mL

Three years later, the patient returned to pursue assisted reproductive technology following marriage. A semen analysis of her husband revealed a sperm concentration of 94 million/mL, with a total motility (progressive and non-progressive) of 15% and strict normal morphology of 2% (Table 2). The DNA fragmentation index (DFI) was 51%. He was diagnosed with asthenoteratozoospermia with high DFI. Given these findings, the couple opted for ICSI. G-IVF PLUS medium (Vitrolife, Gothenburg, Sweden) was used as the fertilization medium. Initially, eight oocytes were thawed, of which four were metaphase II (MII) oocytes, while one degenerated. Another four oocytes were thawed, all in the metaphase I (MI) stage. Seven MI oocytes underwent in vitro meiotic maturation, with five successfully maturing to MII eight hours after thawing. Then fertilization was done with ICSI. From the first batch of four oocytes, one two-pronuclear-stage embryo was obtained; and from the second batch of five oocytes, four two pronuclear-stage embryos were obtained. On day three, the embryo from the first batch was graded as 6C2. Embryos from the second batch were graded as 8C2, 8C4, 7C2, and 6C4. By day five, two blastocysts had formed, both derived from the initially MI oocytes. One blastocyst was graded as 3AA, while the second was an early blastocyst.

**Table 2:** Semen Analysis: Measured and Reference Values

Semen analysis	Obtained values	Reference
Concentration	94x10^6^ per mL	≥15x10^6^ per mL
Total motility	15%	≥40%
Morphology	2%	≥4%
DNA fragmentation index (DFI)	51%	DFI≤15%: Excellent to Good, 15%<dfi average to fair and dfi poor

The 3AA blastocyst was transferred on menstrual cycle day 15 on March 3, 2023, following endometrium preparation. The patient received estradiol valerate (Estrade) 4 mg twice daily from cycle day three to day seven, which was then increased to 6 mg twice daily from cycle day eight to day 14. Transvaginal ultrasound performed before embryo transfer confirmed an endometrial thickness of 12.3 mm. Pregnancy was confirmed 10 days following frozen-thawed embryo transfer with a beta-hCG level of 287 mIU/mL. Clinical pregnancy was verified via transvaginal ultrasound at six-week gestation. The patient received further estradiol valerate (Estrade) 2mg 3# BID, dydrogesterone (Duphaston) 10mg 1# BID, and progesteron gel (Crinone) 90 mg, one tube, BID until 10 weeks of gestation. Non-invasive prenatal testing (NIPT) performed at eight weeks detected a decreased X chromosome signal, with a normalized chromosome value (NCV) of -43.39, raising suspicion of maternal 45,X/46,XX mosaicism. Amniocentesis with microarray analysis later confirmed a normal male karyotype, showing arr(X,Y)x1,(1-22)x2, with a normal male single-nucleotide polymorphism profile. The pregnancy progressed uneventfully, and the patient delivered a healthy male infant by Cesarean section at full term on November 23, 2023 in Canada. The baby weighed 3580 g at birth, and no complications were reported during the delivery.

## Discussion

Since vitrification techniques were recommended for clinical use in 2013, the success rates of oocytes cryopreservation have improved significantly. Preliminary safety data have been reassuring, leading to the removal of oocyte cryopreservation from the experimental category [5]. With more women postponing childbirth, elective oocyte cryopreservation has gained popularity as a potential solution for age-related fertility decline. Nevertheless, several challenges remain, including determining the optimal number of oocytes to store.

A study of 1468 women who underwent elective oocyte cryopreservation for non-oncologic reasons found that pregnancy rates remained age-dependent. The optimal number of stored MII oocytes was at least eight to 10, though this number should be individualized for women over 36 years of age [6]. In this study, a total of 18,915 oocytes were retrieved, with 14,415 MII oocytes selected for vitrification, while 4500 (24%) immature oocytes were discarded. Limited research has explored the fertility potential of these discarded immature oocytes in patients undergoing oocyte cryopreservation.

Two studies have examined the developmental potential of oocytes through rescue in vitro maturation (IVM) compared to their sibling oocytes. While findings have been inconsistent, one study reported signiﬁcantly higher fertilization rates in MII oocytes compared to delayed matured MI-MII oocytes [4,7]. Conversely, another study found comparable fertilization rates between matured oocytes from MI rescue IVM (R-MI) and from germinal vesicle rescue IVM (R-GV) when compared to their sibling MII oocytes [7]. Despite lower blastulation rate in rescue IVM-derived oocytes, both studies reported that once a blastocyst formed, the euploidy rate and “good quality" blastocyst rate were comparable to those derived from in vivo-matured MII oocytes [7]. Additionally, pregnancy, spontaneous pregnancy loss, and live birth rates after single euploid blastocyst transfer showed no statistically signiﬁcant difference between these two groups [4]. A meta-analysis of 24 studies further supported these findings, revealing significantly lower fertilization, cleavage, blastulation, and clinical pregnancy rates in oocytes matured through rescue IVM compared to in vivo-matured MII oocytes. However, no significant differences were found in euploidy or live birth rates following euploid blastocyst transfer [8].

A retrospective cohort study established a cut-off point for the number of mature oocytes required for the routine clinical application of rescue IVM. The study found that having fewer than nine MII oocytes significantly impacted cumulative live birth rates. In patients with fewer than nine MII oocytes, rescue IVM significantly improves clinical pregnancy rate (55.6% vs. 46.7%, P=0.001) and cumulative live birth rates (65.4% vs. 48.1%, P<0.001), whereas no significant benefit was observed in patients with nine or more MII oocytes [9].

Determining the number of oocytes to retrieve in elective cryopreservation cycles remains a challenge, as patients may be uncertain about their future reproductive goals. Furthermore, the quality of retrieved oocytes and subsequent blastocyst formation potential are unknown at the time of retrieval. Whether to discard germinal-vesicle stage or metaphase I oocytes remained an unsolved question.

In this case report, we presented a patient who underwent elective oocyte cryopreservation at the age of 36, retrieving 28 oocytes. Two years later, 12 oocytes were thawed, and a live birth was achieved from an oocyte initially retrieved at the metaphase I stage. After undergoing rescue IVM, the oocyte matured to metaphase II and developed into an embryo graded as 3AA. Given the limited research on the application of rescue IVM in cryopreserved oocytes, this case highlights the developmental potential of immature oocytes in cryopreservation patients and suggests that their utilization may help overall live birth rates.

## Conclusions

In patients undergoing elective oocyte cryopreservation, rescue IVM of immature oocytes may represent a valuable strategy to enhance future live birth potential. The routine practice of discarding metaphase I (MI) oocytes should be reconsidered in light of their possible developmental competence.
